# GABA Regulates Ca^2+^ Oscillations and Synchronization in Pancreatic Beta Cells

**DOI:** 10.3390/metabo16070462

**Published:** 2026-07-01

**Authors:** Vladimir Grubelnik, Marko Marhl

**Affiliations:** 1 Faculty of Electrical Engineering and Computer Science, University of Maribor, Koroška cesta 46, 2000 Maribor, Slovenia; vlado.grubelnik@um.si; 2 Faculty of Natural Sciences and Mathematics, University of Maribor, Koroška cesta 160, 2000 Maribor, Slovenia; 3 Faculty of Medicine, University of Maribor, Taborska ulica 8, 2000 Maribor, Slovenia; 4 Faculty of Education, University of Maribor, Koroška cesta 160, 2000 Maribor, Slovenia

**Keywords:** GABA, pancreatic beta cells, Ca^2+^ oscillations, GABA shunt, ATP dynamics, entrainment, synchronization, mathematical modeling

## Abstract

**Highlights:**

**What are the main findings?**

- GABA regulates the amplitude and frequency of pancreatic beta-cell Ca^2+^ oscillations through both metabolic and paracrine mechanisms.
- The model reproduces Ca^2+^ dynamics in control and GABA-deficient cells and identifies ATP-dependent rhythm generation and GABA-dependent phase adjustment as complementary mechanisms.

**What are the implications of the main findings?**

- Delayed interstitial GABA signaling provides a physiologically plausible mechanism for entrainment and synchronization between non-identical beta cells.
- GABA-mediated phase adjustment and electrical coupling act as complementary mechanisms supporting robust beta-cell synchronization.

**Abstract:**

Background/Objectives: Gamma-aminobutyric acid (GABA) is increasingly recognized as an important modulator of pancreatic beta-cell function, but the mechanisms by which it regulates intracellular Ca^2+^ oscillations and coordinated beta-cell activity remain insufficiently understood. The aim of this study was to investigate how GABA influences the amplitude, frequency, phase adjustment, entrainment, and synchronization of beta-cell Ca^2+^ oscillations. Methods: We developed a reduced ATP–Ca^2+^ oscillation model, based on established beta-cell oscillatory frameworks, and coupled it to the GABA-shunt subsystem derived from our previously established Dual Anaplerotic Model. The model incorporates explicit dynamics of cytosolic Ca^2+^, endoplasmic reticulum Ca^2+^, ATP, and a regulatory variable controlling Ca^2+^ influx, while the interstitial GABA signal is represented as a delayed feedback signal acting on cellular excitability. Single-cell and two-cell simulations were performed to analyze GABA-dependent oscillatory regulation and intercellular coupling. Results: The model reproduced key experimental observations under both control and GABA-deficient conditions, including reduced Ca^2+^-oscillation amplitude and a prolonged oscillation period when GABA production was suppressed. Mechanistically, GABA affected single-cell oscillations through two complementary pathways: metabolically, by modulating ATP production through PEP-related and TCA-related contributions linked to the GABA shunt, and as an interstitial/paracrine signal, by adjusting the phase of Ca^2+^ influx through fast and delayed inhibitory feedback. In the reduced two-cell model, delayed interstitial GABA signaling could phase-lock non-identical oscillators over finite ranges of parameter mismatch. When included as an additional weak effective term, electrical coupling broadened these ranges, consistent with a complementary interaction between GABA-mediated phase adjustment and established electrical coupling. Conclusions: GABA acts as a dual regulator of beta-cell dynamics, linking intracellular metabolism to Ca^2+^-oscillation patterning and promoting coordinated activity through intercellular phase adjustment. The model provides a mechanistic framework connecting GABA metabolism, ATP dynamics, Ca^2+^ signaling, and beta-cell synchronization in pancreatic islets.

## 1. Introduction

Pancreatic beta cells play a central role in glucose homeostasis by coupling metabolic stimulation to insulin secretion, and impairment of this function is a major determinant of diabetes mellitus. In beta cells, nutrient metabolism, ATP production, electrical activity, Ca^2+^ influx, and intracellular Ca^2+^ oscillations are tightly interconnected components of stimulus–secretion coupling. Because pulsatile and coordinated beta-cell activity is essential for efficient insulin release, identifying endogenous regulators of beta-cell Ca^2+^ dynamics remains important for understanding both normal islet physiology and beta-cell dysfunction in diabetes [1].

Among such regulators, gamma-aminobutyric acid (GABA) has attracted attention for several decades. Early work demonstrated that pancreatic beta cells contain unusually high concentrations of GABA, establishing the endocrine pancreas as a major non-neuronal site of GABA accumulation [2]. This concept was extended by the demonstration that glutamic acid decarboxylase (GAD) and GABA colocalize with synaptic-like microvesicles in beta cells, suggesting specialized machinery for GABA storage and secretion [3]. Subsequently, regulated exocytosis of GABA-containing synaptic-like microvesicles was directly demonstrated in pancreatic beta cells [4], and GABA was later shown to function as an autocrine excitatory transmitter in human beta cells through ${GABA}_{A}$ receptor-dependent signaling [5]. Together, these studies established that GABA is not merely a by-product of amino acid metabolism, but an endogenous signaling molecule with functional relevance in pancreatic islets.

In parallel with this signaling perspective, a substantial body of work emphasized the metabolic role of GABA in beta-cell physiology. Broader analyses of amino acid metabolism highlighted the importance of mitochondrial coupling and non-glucose metabolic pathways in insulin secretion [6]. This view was strengthened by the finding that glucose suppresses GABA release from pancreatic beta cells through increased GABA shunt activity [7]. Shortly thereafter, it was shown that glucose-promoted GABA metabolism contributes to insulin secretion and is associated with changes in ATP content and ATP/ADP ratio in beta cells [8]. Additional mechanistic work on glutamate dehydrogenase and amino acid transporters further supported the idea that glutamate/GABA-related pathways are functionally integrated with beta-cell metabolism and secretory activity [9,10]. This metabolic interpretation was explicitly developed in later reviews that argued for a specific role of GABA metabolism and the GABA shunt in sustained insulin secretion and beta-cell bioenergetics [11,12,13].

More recent work brought GABA into the context of oscillatory and collective beta-cell behavior. A major advance came with the demonstration that human beta cells release GABA from cytosolic pools in a pulsatile manner and that this release imposes a synchronizing rhythm on pulsatile insulin secretion, with a critical contribution of the volume-regulated anion channel (VRAC) [14]. More recent reviews have synthesized growing evidence that islet GABA signaling combines receptor-mediated auto/paracrine actions with metabolic coupling through the GABA shunt, particularly in human islets [15,16]. Most importantly for the present study, Ferreira et al. [17] showed that beta-cell-specific loss of GAD65 and GAD67 abolishes endogenous islet GABA and results in abnormal Ca^2+^ oscillations characterized by altered active-phase duration and reduced amplitudes, together with impaired insulin secretion. These findings strongly support the view that endogenous beta-cell-derived GABA is an important determinant of islet Ca^2+^ rhythm generation and coordinated activity.

Despite these advances, the mechanistic basis by which GABA regulates beta-cell Ca^2+^ oscillations remains incompletely understood. In particular, it is still unresolved how GABA-dependent metabolic effects and GABA-dependent modulation of intercellular signaling are translated into quantitative changes in oscillatory frequency, amplitude, entrainment, and the eventual synchronization of beta-cell populations. This unresolved issue is especially important because coordinated oscillatory activity at the islet level is essential for robust pulsatile insulin secretion. In our recent work, we proposed the Dual Anaplerotic Model (DAM), in which the GABA shunt was incorporated as an anaplerotic component of beta-cell metabolism, functionally linked to oscillatory redistribution of mitochondrial and cytosolic metabolites and to ATP generation [18]. That framework now provides a basis for extending the model toward explicit Ca^2+^ dynamics and for interpreting recent experimental observations on GABA-dependent beta-cell behavior.

In the present study, we use an extended mathematical model to investigate how GABA links intracellular metabolism to Ca^2+^ oscillations and coordinated beta-cell dynamics. Building on the DAM framework, we incorporate explicit dynamics of cytosolic Ca^2+^, endoplasmic reticulum Ca^2+^, ATP, and a regulatory variable governing Ca^2+^ influx, while representing interstitial GABA as a delayed interstitial signal that feeds back onto cellular excitability. This formulation enables us to address two interconnected questions. First, we ask how altered GABA production affects the amplitude, frequency, and temporal organization of Ca^2+^ oscillations in a single beta cell, and which underlying mechanisms dominate this response. Second, we ask whether the same GABA-dependent processes can provide an effective coupling pathway between non-identical beta cells and thereby promote entrainment and synchronization.

By combining explicit ATP–Ca^2+^ dynamics with metabolically grounded GABA dynamics and delayed intercellular GABA signaling, the model goes beyond previous conceptual descriptions of GABA action and provides a unified mechanistic framework for interpreting both single-cell and collective beta-cell behavior. In this way, the study not only offers a mechanistic explanation for the experimentally observed effects of GABA deficiency on Ca^2+^ oscillations [17], but also provides a plausible theoretical basis for the synchronizing role of pulsatile beta-cell-derived GABA within the islet [14,15]. Overall, our findings support the view that GABA is an important regulator of beta-cell oscillatory dynamics and collective activity, acting through a functional link between mitochondrial metabolism, ATP production, Ca^2+^ influx, and intercellular phase coordination.

To clarify the conceptual scope of the present study, the novelty of our model does not lie in proposing a new general mechanism for beta-cell ATP-Ca^2+^ oscillations, which have been extensively studied in previous theoretical frameworks. Instead, we use established ATP-Ca^2+^ oscillator concepts as the dynamical backbone and introduce a GABA-centered extension derived from the DAM. In this formulation, GABA is not treated as an externally imposed signal or as an isolated metabolite, but as a dynamically generated variable that feeds back onto the ATP-Ca^2+^ oscillator through two complementary mechanisms. First, DAM-derived GABA-related fluxes modulate PEP-related and TCA-related ATP production, thereby linking GABA-shunt metabolism to ATP-dependent rhythm shaping. Second, released interstitial GABA acts as a delayed feedback signal on Ca^2+^ influx, providing a mechanism for phase adjustment, entrainment, and synchronization between non-identical beta-cell oscillators. Thus, the conceptual advance of the present work is the integration of metabolically grounded GABA dynamics into an established ATP-Ca^2+^ oscillatory framework, allowing GABA to be analyzed as a dynamic regulator of both single-cell oscillation patterning and intercellular coordination.

## 2. Materials and Methods

The mathematical model developed in this study is based on established frameworks of intracellular Ca^2+^ dynamics and incorporates key mechanisms described in previous models, such as the Integrated Oscillator Model (IOM) [19]. These mechanisms are reduced to a minimal formulation that preserves the essential oscillatory behavior arising from Ca^2+^ exchange between the cytosol and the endoplasmic reticulum, in line with classical minimal models of calcium oscillations [20,21,22]. In addition, the model explicitly incorporates coupling between Ca^2+^ and metabolic dynamics, thereby reproducing the characteristic phase relationship between Ca^2+^ and metabolic/ATP oscillations reported in previous studies [19,22,23]. Within this framework, the primary aim of the model is to introduce GABA-dependent processes in order to investigate how GABA modulates Ca^2+^ oscillations, their phase relationships, and synchronization in pancreatic beta cells.

Accordingly, the present model should be viewed as a GABA-centered extension of established beta-cell ATP-Ca^2+^ oscillation models, rather than as a replacement for detailed electrophysiological or integrated oscillator models. The reduced ATP-Ca^2+^ framework is used here to provide the oscillatory backbone, whereas the DAM-derived GABA subsystem is introduced to examine how GABA metabolism and delayed interstitial GABA signaling modulate oscillation amplitude, frequency, phase relationships, entrainment, and synchronization.

Because the present study focuses on slow, minute-scale Ca^2+^ oscillations and their GABA-dependent phase adjustment, the model does not explicitly resolve fast membrane-potential dynamics or individual ion-channel currents and gating dynamics. Instead, membrane excitability is represented in an effective form through the Ca^2+^ influx term and the associated activation variable. This coarse-grained representation summarizes the net effect of ATP-dependent K_ATP_-channel closure, membrane depolarization, VGCC opening, calcium-dependent inactivation, and GABA receptor-mediated modulation of Ca^2+^ entry. Such a reduction is appropriate for the aims of the present study, because the model is intended to reproduce slow changes in Ca^2+^ oscillation amplitude, frequency, phase entrainment, and synchronization, rather than spike-by-spike electrical bursting or detailed receptor/channel kinetics. At the same time, this formulation avoids introducing a large number of poorly constrained electrophysiological parameters while retaining the experimentally supported delayed negative feedback by which beta-cell-derived GABA modulates Ca^2+^ influx.

We first introduce a Single-Cell Model, which describes intracellular Ca^2+^ dynamics within an individual beta cell. The model integrates metabolic and electrophysiological components, with particular emphasis on the role of GABA in mitochondrial ATP production via the anaplerotic GABA shunt. In addition, GABA release is incorporated as a dynamic process that introduces delayed negative feedback on Ca^2+^ influx, thereby modulating the temporal characteristics of intracellular Ca^2+^ oscillations.

The framework is subsequently extended to a Two-Cell Coupled Model, in which individual cellular oscillators are interconnected through GABA-mediated coupling. This formulation enables us to examine how the coupling between oscillatory Ca^2+^ dynamics and oscillatory GABA signaling at the single-cell level can facilitate phase alignment and promote synchronization of Ca^2+^ oscillations across cells.

### 2.1. Single-Cell Model

**Figure 1:**
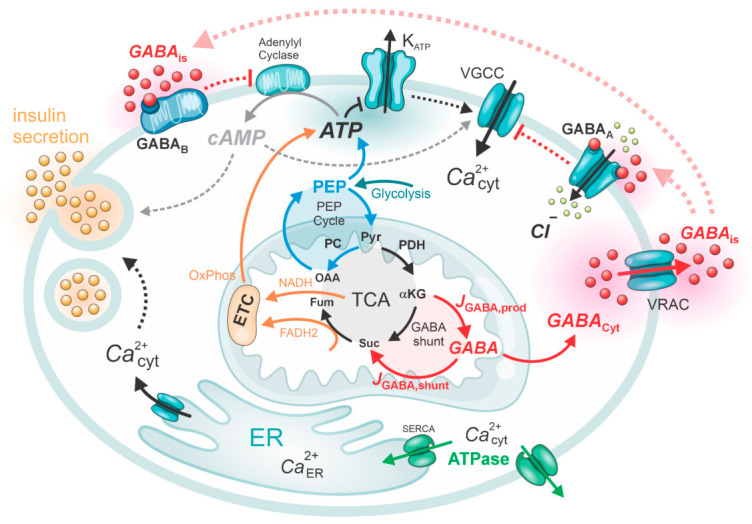
Schematic representation of the cellular processes influencing intracellular Ca^2+^ oscillatory dynamics in a single beta cell. The individual fluxes and regulatory influences are described in detail in the main text. Dashed arrows indicate signaling or regulatory interactions. Solid red arrows denote the intracellular GABA metabolic pathway, including GABA production and the GABA shunt, whereas dashed red arrows denote interstitial GABA-mediated autocrine signaling through GABA receptors. Abbreviations: αKG—α-ketoglutarate; ATP—adenosine triphosphate; ATPase—ATP-consuming Ca^2+^ pumps; cAMP—cyclic adenosine monophosphate; ${Ca}_{cyt}^{2+}$—cytosolic calcium; ${Ca}_{ER}^{2+}$—endoplasmic reticulum calcium; Cl^−^—chloride ion; ER—endoplasmic reticulum; ETC—electron transport chain; FADH_2_—reduced flavin adenine dinucleotide; Fum—fumarate; GABA—γ-aminobutyric acid; ${GABA}_{cyt}$—cytosolic GABA; ${GABA}_{is}$—interstitial GABA; ${GABA}_{A}$—${GABA}_{A}$ receptor; ${GABA}_{B}$—${GABA}_{B}$ receptor; $J_{GABA,prod}$—GABA production flux; $J_{GABA,shunt}$—GABA shunt flux; $K_{ATP}$—ATP-sensitive potassium channel; NADH—reduced nicotinamide adenine dinucleotide; OAA—oxaloacetate; OxPhos—oxidative phosphorylation; PC—pyruvate carboxylase; PDH—pyruvate dehydrogenase; PEP—phosphoenolpyruvate; Pyr—pyruvate; SERCA—sarco/endoplasmic reticulum Ca^2+^-ATPase; Suc—succinate; TCA—tricarboxylic acid cycle; VGCC—voltage-gated calcium channel; VRAC—volume-regulated anion channel.

The cellular processes governing intracellular Ca^2+^ oscillations in a single β-cell are schematically illustrated in Figure 1. The model includes Ca^2+^ fluxes between the cytosol and the endoplasmic reticulum (ER), as well as Ca^2+^ influx across the plasma membrane through voltage-gated Ca^2+^ channels (VGCCs). VGCC opening is regulated by the membrane potential, such that depolarization promotes Ca^2+^ entry. Membrane depolarization is, in turn, favored by closure of ATP-sensitive K^+^ channels (K_ATP_ channels), driven by an increase in local ATP concentration near the plasma membrane. This local ATP signal is associated with PEP-cycle activity and ATP delivery to the microdomain surrounding K_ATP_ channels, whereas mitochondrial oxidative phosphorylation provides the principal energetic background for this process [23,24]. Within this framework, ATP production is additionally influenced by the GABA shunt, which acts as an anaplerotic pathway [18] feeding succinate into the TCA cycle and thereby reinforcing mitochondrial oxidative metabolism. In this manner, GABA metabolism contributes to ATP generation and functionally couples mitochondrial metabolism to the regulation of Ca^2+^ dynamics. The model further includes net cytosolic GABA production (${GABA}_{cyt}$), as described in detail in our previous study [18], as well as its release into the interstitial space through VRAC, thereby generating the interstitial GABA signal ${GABA}_{is}$. Interstitial GABA is assumed to inhibit Ca^2+^ influx through mechanisms involving both ${GABA}_{A}$ and ${GABA}_{B}$ receptors [17], as will be described in more detail below. In effective form, these pathways are represented in the model as a delayed negative-feedback loop through which GABA release suppresses Ca^2+^ entry and modulates the temporal characteristics of intracellular Ca^2+^ oscillations.

#### 2.1.1. Ca^2+^ Dynamics

Intracellular Ca^2+^ dynamics is described by the following two differential equations:

(1) $$\frac{d{Ca}_{cyt}}{dt}=J_{in}-J_{out}+J_{ER,leak}-J_{ER,pump}+J_{ER,ch}\;,$$

(2) $$\frac{d{Ca}_{ER}}{dt}=J_{ER,pump}-J_{ER,leak}-J_{ER,ch}\;,$$

which represent the temporal evolution of Ca^2+^ concentration in the cytosol (${Ca}_{cyt}$) and in the endoplasmic reticulum (${Ca}_{ER}$), respectively. The model accounts for Ca^2+^ exchange between the cytosol and the ER, as well as Ca^2+^ fluxes across the plasma membrane.

In the following, the individual flux terms are described in more detail. The values of the model constants given below represent reference values in arbitrary units.

Within this framework, the flux $J_{ER,pump}$ represents the active uptake of Ca^2+^ from the cytosol into the ER, mediated by the sarco/endoplasmic reticulum Ca^2+^-ATPase (SERCA). In the present model, this flux is described by

(3) $$J_{ER,pump}=k_{ER,pump}\cdot {Ca}_{cyt}\;,$$

where $k_{ER,pump}=4$, assuming a linear dependence of pump activity on cytosolic Ca^2+^ concentration. In this formulation, the dependence of the flux on ATP concentration is neglected, as its variations in the cytosol are relatively small. This simplification is justified by experimental evidence showing that cytosolic ATP levels exhibit relatively small fluctuations compared to those in the submembrane compartment [25] and in the microdomain associated with $K_{ATP}$ channels, where local ATP dynamics are sufficient to regulate channel permeability [23]. Instead, the model explicitly incorporates Ca^2+^ dependence, as SERCA activity is tightly regulated by cytosolic Ca^2+^ levels. An increase in $Ca_{cyt}$ enhances Ca^2+^ binding to the pump, thereby increasing its turnover rate and facilitating Ca^2+^ sequestration into the ER. Capturing this dependence is essential for accurately reproducing intracellular Ca^2+^ dynamics, particularly the feedback mechanisms governing Ca^2+^ oscillations and ER loading.

The flux $J_{ER,leak}$ describes passive Ca^2+^ leak from the ER back into the cytosol, reflecting the basal permeability of the ER membrane. In the present model, this flux is described by

(4) $$J_{ER,leak}=k_{ER,leak}\cdot {Ca}_{ER}\;,$$

where $k_{ER,leak}=0.5$, and the leak rate is assumed to increase linearly with the ER luminal Ca^2+^ concentration. This assumption reflects the fact that passive Ca^2+^ efflux from the ER depends on the ER Ca^2+^ load, with higher $Ca_{ER}$ generating a larger concentration gradient and thereby a greater leak flux into the cytosol. Notably, the dependence of the flux on the concentration difference between the ER and the cytosol is not modeled explicitly. This simplification is justified by the fact that Ca^2+^ concentration in the ER is several orders of magnitude higher than in the cytosol. Consequently, the concentration gradient effectively follows variations in $Ca_{ER}$, which supports the approximation of the leak flux as being proportional to $Ca_{ER}$.

The term $J_{ER,ch}$ represents Ca^2+^ release from the ER through Ca^2+^-permeable ER channels and accounts for channel-mediated mobilization of stored Ca^2+^ into the cytosol. In the present model, this flux is described by

(5) $$J_{ER,ch}=k_{ER,ch}\cdot \frac{{Ca}_{cyt}^{n_{ch}}}{K_{ch}^{n_{ch}}+{Ca}_{cyt}^{n_{ch}}}\cdot {Ca}_{ER}\;,$$

with $k_{ER,ch}=1$, $K_{ch}=0.4$, and $n_{ch}=4$. This formulation assumes a sigmoidal dependence of channel activation on cytosolic Ca^2+^ concentration, reflecting the cooperative nature of Ca^2+^-induced Ca^2+^ release (CICR). The multiplicative dependence on ${Ca}_{ER}$ accounts for the fact that the magnitude of Ca^2+^ release is also governed by the ER Ca^2+^ load, with higher luminal Ca^2+^ levels providing a stronger driving force for Ca^2+^ efflux.

In addition to intracellular Ca^2+^ exchange, the model also includes Ca^2+^ transport across the plasma membrane, represented by the fluxes $J_{in}$ and $J_{out}$. The efflux term $J_{out}$ describes Ca^2+^ extrusion from the cytosol to the extracellular space via plasma membrane Ca^2+^ clearance mechanisms, predominantly Ca^2+^-ATPases and related outward transport processes. In the present model, this flux is described by

(6) $$J_{out}=k_{out}\cdot Ca_{cyt}\;,$$

with $k_{out}=1$, assuming a linear dependence of Ca^2+^ extrusion on cytosolic Ca^2+^ concentration. The proportional dependence on $Ca_{cyt}$ captures the first-order approximation of Ca^2+^ extrusion kinetics, where the rate of transport increases with substrate availability. This is consistent with the behavior of plasma membrane Ca^2+^ pumps and exchangers, which respond to elevated cytosolic Ca^2+^ by enhancing Ca^2+^ clearance, thereby contributing to the restoration of basal intracellular Ca^2+^ levels.

The influx term $J_{in}$ denotes Ca^2+^ entry from the extracellular space into the cytosol, primarily through voltage-gated Ca^2+^ channels (VGCCs). Accordingly, $J_{in}$ should be interpreted as an effective, time-averaged Ca^2+^ influx term relevant for slow Ca^2+^ oscillations, rather than as a detailed conductance-based description of membrane voltage and individual VGCC gating dynamics. In the present model, this flux is described by

(7) $$J_{in}=g_{in}\;x\;\frac{K_{in}}{K_{in}+{Ca}_{cyt}},$$

where $g_{in}$ represents the maximum influx rate modulated by intracellular signaling, and x denotes the channel activation variable. In the model, we additionally include an inhibitory dependence of the influx term $J_{in}$ on $Ca_{cyt}$, thereby phenomenologically representing calcium-dependent inactivation (CDI) of voltage-gated Ca^2+^ channels. In this way, an increase in intracellular Ca^2+^ reduces channel availability and thereby limits further Ca^2+^ influx [26,27]. Here, $K_{in}=0.2$ denotes the half-saturation constant controlling the dependence of the influx on cytosolic Ca^2+^.

The parameter $g_{in}$ is further defined as

(8) $$g_{in}=g_{in,0}-k_{in,G}\cdot {GABA}_{is}\;,$$

where $g_{in,0}=8$ is the baseline maximal ${Ca}^{2+}$ influx rate in the absence of inhibitory GABAergic input, and $k_{in,G}=500$ quantifies the strength by which interstitial GABA suppresses ${Ca}^{2+}$ influx. Here, ${GABA}_{is}$ denotes the interstitial GABA concentration, which follows the intracellular GABA concentration with a time delay, as described in more detail below. In this way, we assume that the delayed interstitial GABA signal modulates Ca^2+^ influx via a rapid inhibitory feedback loop. This term provides a phenomenological description of the fast ionotropic action of interstitial GABA on beta-cell excitability and membrane-potential-dependent Ca^2+^ entry, consistent with the current view that GABA released from beta cells acts through both ${GABA}_{A}$ and ${GABA}_{B}$ receptors and that exogenous GABA suppresses islet Ca^2+^ oscillations through a mechanism involving both receptor classes [15,17].

In modeling the influx $J_{in}$ (Equation (7)), we additionally introduce a slower inhibitory feedback loop via the regulatory variable x. The gating variable x follows first-order kinetics,

(9) $$\frac{dx}{dt}=\frac{x_{\infty ,G}-x}{\tau_{x}}\;,$$

where $\tau_{x}=1$ determines the timescale of adaptation of the gating process. The steady-state value $x_{\infty ,G}$ depends on both ATP and GABA levels,

(10) $$x_{\infty ,G}=\{\begin{array}{cc} x_{\infty ,0}-k_{x,G}\;{GABA}_{is}; & ATP>{ATP}_{open}\\ 0; & ATP<{ATP}_{open} \end{array}\;,$$

where $x_{\infty ,0}=1$ is the baseline steady-state activation level in the absence of GABA, and $k_{x,G}=100$ determines the strength of GABA-dependent inhibition of channel activation. Since x positively regulates the influx $J_{in}$ (Equation (7)), a reduction in $x_{\infty ,G}$ mediated by ${GABA}_{is}$ (Equation (10)) implies that delayed interstitial GABA gradually reduces the effective drive for Ca^2+^ influx on a slower timescale. Physiologically, this slow inhibitory component is intended to phenomenologically represent a metabotropic ${GABA}_{B}$-like pathway acting on slower excitability and adaptation processes, consistent with inhibitory G-protein (Gi/o)-dependent inhibition of adenylyl cyclase and the resulting reduction in Ca^2+^ influx [15]. Together, these terms (Equations (7)–(10)) define a physiologically plausible delayed negative feedback loop through which interstitial GABA (${GABA}_{is}$) can suppress excessive Ca^2+^ entry [17]. The parameter ${ATP}_{open}=0.65$ represents the threshold ATP concentration above which Ca^2+^ channels can become active (open), whereas below this threshold the channels remain closed.

In this formulation, the two GABA-dependent inhibitory terms should be interpreted as effective representations of the net influence of interstitial GABA on Ca^2+^ entry, rather than as receptor-specific kinetic descriptions. Thus, the model does not assign distinct microscopic rate laws to ${GABA}_{A}$ and ${GABA}_{B}$ receptor activation, chloride conductance, membrane-potential changes, Gi/o signaling, or cAMP-dependent downstream pathways. Instead, these processes are condensed into fast and slower inhibitory components acting on the effective Ca^2+^ influx term, which is the level of description required for the present analysis of slow, minute-scale Ca^2+^ oscillations and phase adjustment. Consequently, the model predictions should be interpreted in terms of the net GABA-dependent modulation of Ca^2+^-oscillation amplitude, timing, and synchronization, rather than in terms of receptor-specific electrical or biochemical kinetics.

#### 2.1.2. ATP Dynamics and Coupling to the DAM

In the present model, ATP dynamics is described at the level of the submembrane ATP pool, rather than bulk cytosolic ATP, because ATP oscillations in the submembrane region are substantially more pronounced and are more directly relevant for the regulation of Ca^2+^ influx and K_ATP_-dependent excitability than ATP in the bulk cytosol [23]. The temporal evolution of ATP is therefore described by:

(11) $$\frac{dATP}{dt}=J_{ATP,prod}-J_{ATP,pump}-J_{ATP,use},$$

where $J_{ATP,prod}$ denotes ATP production, $J_{ATP,pump}$ represents ATP consumption by Ca^2+^-ATPases, and $J_{ATP,use}$ accounts for other ATP-consuming cellular processes.

The ATP production term is written as

(12) $$J_{ATP,prod}=\frac{v_{PEP}}{1+{\left(\frac{{Ca}_{cyt}}{K_{Ca}}\right)}^{n}}+v_{TCA}\cdot {Ca}_{cyt},$$

where $K_{Ca}=0.2$ is the half-saturation constant controlling the inhibitory effect of cytosolic Ca^2+^ on the PEP-dependent component of ATP production, and n=4 is the corresponding Hill coefficient determining the steepness of this dependence. Equation (12) separates ATP production into two physiologically distinct components. The first term describes ATP production associated with the cataplerotic phase and the associated PEP-cycle activity, whereas the second term represents the oxidative mitochondrial contribution associated with TCA-cycle activity.

The inverse dependence of the first term on $Ca_{cyt}$ is an essential feature of the model. It reflects the idea that ATP production via the PEP cycle is most effective when cytosolic Ca^2+^ is low, i.e., during the cataplerotic phase, when carbon is preferentially routed through pyruvate carboxylase (PC) rather than pyruvate dehydrogenase (PDH), and when PEP cycling becomes an important source of ATP delivery to the submembrane region [18,23]. In this regime, the availability of carbon skeletons for PEP cycling depends strongly on the anaplerotic input provided by the GABA shunt. Within the DAM framework [18], this cataplerotic contribution is associated with the flux $J_{13}$, which describes carbon redistribution toward the GABA-related pool during the cataplerotic phase of the oscillation cycle. In the present formulation, this flux is identified with the effective GABA production flux, $J_{GABA,prod}\equiv J_{13}$. Accordingly, the effective strength of the PEP-dependent ATP-producing branch is written as

(13) $$v_{PEP}=v_{PEP,0}+k_{PEP,G}\cdot J_{GABA,prod},$$

where $v_{PEP,0}=0.1$ is the basal contribution of the PEP-related ATP-producing component, and $k_{PEP,G}=1$ determines how strongly this component is enhanced by the cataplerotic flux $J_{GABA,prod}=J_{13}$. In this way, the model links GABA-dependent cataplerotic carbon redistribution directly to the PEP-cycle-associated ATP production that is most effective during the low-Ca^2+^ phase of the oscillation.

The second term in Equation (12), $v_{TCA}\cdot Ca_{cyt}$, represents the oxidative component of ATP production. This term captures the fact that mitochondrial ATP synthesis in the submembrane region is also stimulated during the oxidative phase, when elevated cytosolic Ca^2+^ promotes mitochondrial metabolism, in part through activation of PDH and enhanced TCA-cycle turnover. In the DAM framework, this oxidative contribution is linked to the GABA shunt flux $J_{32}$, which describes the return of carbon from the GABA-related pool back into the left part of the TCA cycle, thereby reinforcing oxidative ATP production during the phase in which fresh carbon enters the cycle through GABA-shunt-dependent anaplerosis [18]. In the present formulation, this flux is identified with the effective GABA-shunt contribution, $J_{GABA,shunt}\equiv J_{32}$. We therefore write

(14) $$v_{TCA}=v_{TCA,0}+k_{TCA,G}\cdot J_{GABA,shunt},$$

where $v_{TCA,0}=1$ is the basal oxidative contribution to ATP production, and $k_{TCA,G}=2$ quantifies the extent to which this component is increased by the flux $J_{GABA,shunt}=J_{32}$. Thus, the model explicitly links GABA-shunt-mediated return of carbon into the TCA cycle to the oxidative ATP-producing branch that predominates during the high-Ca^2+^ phase of the oscillation.

A key point is that the fluxes $J_{13}$ and $J_{32}$, as well as the dynamics of cytosolic GABA, are not introduced here as ad hoc functions, but are taken directly from the previously published DAM formulation [18]. In the original DAM study, the time courses of $Ca_{cyt}$ and ATP were prescribed from experimental fits, whereas the remaining metabolic pools and inter-pool fluxes were modeled explicitly. In the present study, this logic is extended one step further: the same DAM subsystem is retained for the calculation of $J_{13}$, $J_{32}$, and ${GABA}_{cyt}$, but $Ca_{cyt}$ and ATP are now computed self-consistently from the differential equations of the present model. Thus, the DAM provides the internal metabolic structure required to determine how GABA-shunt-dependent carbon redistribution feeds back onto ATP production and, indirectly, onto Ca^2+^ dynamics.

ATP consumption by Ca^2+^-transporting ATPases is described by

(15) $$J_{ATP,pump}=k_{pump}\cdot {Ca}_{cyt},$$

where $k_{pump}=1.5$ denotes the effective rate constant for ATP consumption by Ca^2+^-ATPases. This term represents the ATP cost of Ca^2+^ sequestration and extrusion. In this effective formulation, ATP consumption is assumed to increase linearly with cytosolic Ca^2+^, reflecting the enhanced activity of Ca^2+^-ATPases when intracellular Ca^2+^ levels are elevated.

Finally, we include a further ATP consumption term,

(16) $$J_{ATP,use}=k_{use}\cdot ATP,$$

where $k_{use}=0.1$ denotes the effective rate constant for ATP consumption by other ATP-dependent cellular processes that are not modeled explicitly. These include, for example, ATP utilization in biosynthetic reactions, maintenance processes, cyclic nucleotide metabolism, and broader inhibitory effects associated with high ATP availability, including feedback on metabolic enzymes. This term therefore represents a generic ATP sink that prevents unbounded ATP accumulation and contributes to shaping the oscillatory ATP profile.

Together, Equations (11)–(16) define the ATP dynamics of the present single-cell model. Relative to the original DAM formulation, the essential novelty here is that ATP is no longer prescribed externally, but emerges dynamically from the interplay between Ca^2+^-dependent ATP consumption, PEP-cycle-associated ATP production, oxidative ATP production, and the metabolic redistribution of carbon through the GABA shunt. This explicit treatment of ATP dynamics is crucial, because ATP acts as the central link between mitochondrial metabolism, GABA-shunt activity, and Ca^2+^-dependent excitability in the present model.

#### 2.1.3. DAM-Based GABA Dynamics

To describe GABA dynamics in a metabolically consistent way, we retained the DAM subsystem introduced in our previous study [18], including the same pool variables $P_{0}$, $P_{1}$, $P_{2}$, and $P_{3}$, as well as the same inter-pool fluxes $J_{0}$, $J_{01}(=J_{21})$, $J_{12}$, $J_{13}$, and $J_{32}$. In the original DAM formulation, these equations were used together with experimentally fitted time courses of $Ca_{cyt}$ and ATP. In the present study, however, $Ca_{cyt}$ and ATP are no longer prescribed externally, but are computed self-consistently from the differential equations introduced above. Thus, the DAM subsystem is retained in its original form, whereas its coupling to the rest of the model is extended by replacing the previously imposed ${Ca}_{cyt}$ and ATP dynamics with explicitly calculated variables.

Within this framework, the variable $P_{3}$, representing the GABA pool in the original DAM, directly provides the cytosolic GABA dynamics used in the present study. Accordingly, cytosolic GABA is not introduced here through an additional phenomenological equation, but follows directly from the original DAM equations. This preserves the metabolic interpretation of GABA as an integral part of the oscillatory redistribution of carbon between the glycolytic pool, the two TCA-cycle-related pools, and the GABA pool, as described previously [18].

As in the DAM, the flux $J_{13}$ represents the cataplerotic branch associated with transfer from the right half of the TCA cycle toward the GABA pool, whereas $J_{32}$ represents the oxidative return flux from the GABA pool back toward the left half of the TCA cycle. These two fluxes are of particular importance in the present model because they provide the metabolic link between the DAM subsystem and ATP production. Specifically, $J_{13}$ modulates the PEP-cycle-related contribution to ATP production, whereas $J_{32}$ modulates the oxidative TCA-related contribution, as introduced in Equations (13) and (14). In this way, the previously developed DAM structure is preserved, while its metabolic output is now dynamically coupled to the explicitly modeled ATP and Ca^2+^ oscillations.

The remaining DAM equations and parameter values are the same as in the original publication [18] and are therefore not repeated here in full. This approach avoids unnecessary duplication while maintaining full consistency with the previously established metabolic framework. The essential extension introduced in the present study is that the DAM subsystem now operates within a closed dynamical model in which ${Ca}_{cyt}$, ${Ca}_{ER}$, ATP, and GABA mutually interact. Consequently, GABA dynamics is no longer driven by externally imposed oscillatory inputs, but emerges from the coupled metabolic-calcium system itself.

To account for the fact that interstitial GABA does not follow cytosolic GABA instantaneously, the interstitial GABA concentration $GABA_{is}$ is modeled as a delayed function of cytosolic GABA,

(17) $${GABA}_{is}(t)=r_{G}{GABA}_{cyt}(t-\tau_{G}),$$

where $r_{G}=0.01$ denotes the proportionality factor relating cytosolic and interstitial GABA levels, and $\tau_{G}=1$ represents a short effective delay associated with GABA release, diffusion in the interstitial space, and uptake/clearance. The introduction of the factor $r_{G}<1$ is physiologically motivated by the fact that beta cells maintain a high intracellular GABA pool, estimated to be in the millimolar range, whereas interstitial GABA in the islet is thought to lie in the nanomolar-to-low-micromolar range [14,15,16]. At present, however, a quantitative phase delay between cytosolic and interstitial GABA oscillations in pancreatic beta cells has not been experimentally established. Available evidence indicates that human beta cells release GABA directly from a cytosolic pool via VRAC in a pulsatile manner, with secretion periods typically in the 4–10 min range, while taurine transporter (TauT)-mediated uptake contributes to maintaining low interstitial GABA levels; together, these findings support a rapid release-clearance cycle but do not provide a direct numerical estimate of the lag between intracellular and interstitial GABA dynamics [14,15,16]. Recent work further showed that endogenous beta-cell-derived GABA is required for proper islet Ca^2+^ oscillation dynamics and suggested that GABA signaling may operate as a delayed negative feedback mechanism, yet the magnitude of this delay has likewise not been measured directly [17]. Accordingly, in the absence of direct experimental constraints, interstitial GABA is represented here by a small effective delay.

### 2.2. Two-Cell Coupled Model

**Figure 2:**
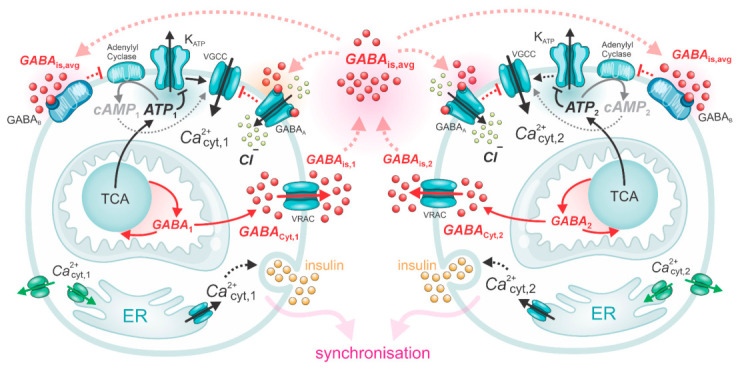
Schematic representation of GABA-mediated intercellular coupling between two beta cells and its role in the synchronization of intracellular Ca^2+^ oscillations. The individual labels are defined in Figure 1. Dashed arrows indicate signaling or regulatory interactions. Solid red arrows denote the GABA metabolic pathway and GABA release toward the interstitial space, whereas dashed red arrows denote interstitial GABA-mediated paracrine/autocrine signaling through GABA receptors. In addition, ${GABA}_{avg}$ is introduced here as the average interstitial GABA signal shared by the coupled cells. Through ${GABA}_{A}$ and ${GABA}_{B}$ receptor-mediated pathways, this common interstitial GABA signal exerts an inhibitory effect on Ca^2+^ influx into each cell, thereby modulating intracellular Ca^2+^ signals and promoting synchronized intracellular activity across the coupled system.

To demonstrate how GABA promotes coordinated activity between pancreatic beta cells, we extended the single-cell model to a system of two coupled cells (Figure 2). Each cell retained the same intracellular dynamics as in the single-cell case, including Ca^2+^ handling, ATP production, and GABA oscillations, while coupling was introduced through a shared interstitial GABA signal. In this way, intracellular metabolic oscillations are converted into an intercellular coupling signal capable of entraining Ca^2+^ dynamics between neighboring beta cells.

The common interstitial GABA signal is defined as the average value of the interstitial GABA level associated with each individual cell. The arithmetic mean of the interstitial GABA level of the two-cell system is given by

(18) $${GABA}_{is,avg}=\frac{1}{N}\sum_{i=1}^{N}{GABA}_{is,i},\;N=2,$$

where ${GABA}_{is,i}=r_{G}{GABA}_{cyt,i}(t\;-\;\tau_{G})$ denotes the interstitial GABA signal associated with the i-th cell (see Equation (17)).

As illustrated schematically in Figure 2, the shared interstitial GABA signal, ${GABA}_{is,avg}$, acts on both neighboring cells through ${GABA}_{A}$ and ${GABA}_{B}$ receptor-mediated pathways. Through these interactions, the common interstitial GABA pool modulates Ca^2+^ influx into each cell and thereby influences the timing and amplitude of intracellular Ca^2+^ oscillations. Because both cells are exposed to the same interstitial inhibitory GABA signal, this coupling provides a mechanism for phase alignment of their Ca^2+^ dynamics. In this way, GABA-mediated intercellular coupling can promote synchronization of Ca^2+^ signals in two-cell as well as multicellular systems, thereby supporting coordinated pulsatile insulin secretion at the islet level.

## 3. Results

The mathematical model defined by Equations (1)–(18) was used to investigate how GABA regulates Ca^2+^ oscillations and coordinated beta-cell dynamics. We begin by analyzing the consequences of altered GABA production, since this question is directly motivated by recent experimental observations showing that reduced endogenous GABA availability leads to decreased Ca^2+^ oscillation amplitude and prolonged oscillatory periods in pancreatic beta cells [17]. The first part of the Section 3 therefore examines how changes in GABA production affect the amplitude, frequency, and temporal organization of cytosolic Ca^2+^ oscillations in a single beta cell. We then dissect the underlying mechanisms by separating the metabolic contribution of GABA to ATP production from its interstitial/paracrine signaling effect on Ca^2+^ influx, thereby identifying the dominant processes responsible for the observed oscillatory phenotype. Finally, we extend the analysis to coupled cells and show how delayed interstitial GABA signaling provides a physiologically plausible pathway for phase adjustment, entrainment, and synchronization of beta-cell oscillations. In this way, this section proceeds from experimentally motivated single-cell effects of altered GABA production to the emergence of coordinated multicellular dynamics.

### 3.1. GABA Controls Amplitude and Frequency of Ca^2+^ Oscillations in a Single Beta Cell

Within the proposed single-cell framework, we first examine how GABA production modulates intracellular Ca^2+^ oscillations. This question is directly motivated by the experimental findings of Ferreira et al. [17], who showed that endogenous beta-cell-derived GABA is essential for maintaining properly shaped Ca^2+^ oscillations, whereas loss of GABA signaling leads to a reduced oscillation amplitude and a prolonged oscillation period.

To investigate this effect, we varied the relative strength of the flux representing GABA production. Specifically, we introduced the dimensionless parameter $p_{GABA,prod}\in [0,1]$, which rescales the DAM flux $J_{13}$ [18]. In the present model, $J_{13}$ is identified with the effective GABA production flux, $J_{GABA,prod}$, such that

(19) $$J_{GABA,prod}\;:\;=\;p_{GABA,prod}\;J_{GABA,prod}.$$

**Figure 3:**
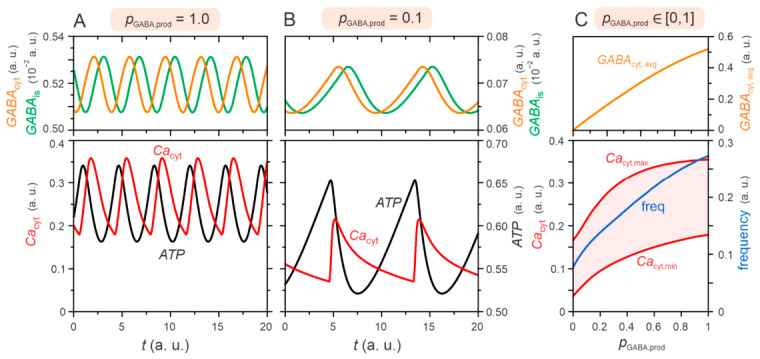
Effect of GABA production on single-cell oscillatory dynamics. (**A**) Reference dynamics for $p_{GABA,prod}=1.0$, showing intracellular ${GABA}_{cyt}$, interstitial ${GABA}_{is}$, cytosolic calcium ${Ca}_{cyt}$, and local ATP near $K_{ATP}$ channels. (**B**) Modified dynamics under reduced GABA production, $p_{GABA,prod}=0.1$. (**C**) Dependence of the average intracellular GABA concentration, ${GABA}_{cyt,avg}$, the minimum and maximum values of the calcium oscillations, ${Ca}_{cyt,min}$ and ${Ca}_{cyt,max}$, and the oscillation frequency on $p_{GABA,prod}$.

Here, $p_{GABA,prod}=0$ corresponds to complete suppression of GABA production, whereas $p_{GABA,prod}=1$ retains the original DAM flux unchanged. In this way, $p_{GABA,prod}$ provides a direct control parameter for intracellular GABA generation. Because cytosolic GABA in turn determines both the metabolic contribution of GABA to ATP production and the delayed interstitial GABA signal, varying $p_{GABA,prod}$ alters the coupled dynamics of GABA, ATP, and Ca^2+^ oscillations, as illustrated in Figure 3.

Figure 3A shows the model dynamics for the reference condition, $p_{GABA,prod}=1.0$, for which GABA production remains unchanged relative to the original DAM formulation. The upper panel displays oscillations of intracellular GABA, ${GABA}_{cyt}$, together with the corresponding interstitial GABA signal, $GABA_{is}$. As expected from Equation (17), ${GABA}_{is}$ follows the intracellular oscillations with a small delay and reaches concentrations that are approximately two orders of magnitude lower than those of ${GABA}_{cyt}$. The lower panel shows the corresponding oscillations of cytosolic calcium, ${Ca}_{cyt}$, and local ATP concentration near $K_{ATP}$ channels, illustrating the coupled metabolic and signaling dynamics under reference conditions.

Figure 3B shows the corresponding model dynamics under strongly reduced GABA production, $p_{GABA,prod}=0.1$. Under these conditions, both intracellular and interstitial GABA concentrations are substantially reduced relative to the reference case. This reduction is accompanied by pronounced changes in Ca^2+^ dynamics. In particular, the amplitudes of ${Ca}_{cyt}$ oscillations are reduced and the oscillation frequency decreases, corresponding to a prolongation of the oscillation period. Thus, diminished GABA production leads to both weaker and slower cytosolic Ca^2+^ oscillations. These predictions are in good qualitative agreement with the experimental observations of Ferreira et al. [17], who reported that impaired endogenous GABA signaling is associated with reduced Ca^2+^ oscillation amplitude and prolonged oscillatory periods.

Figure 3C summarizes the dependence of the model output on $p_{GABA,prod}$. The upper panel shows that the average intracellular GABA concentration, ${GABA}_{cyt,avg}$, increases monotonically with increasing GABA production. The lower panel shows the corresponding changes in Ca^2+^ oscillatory behavior. Both the minimum and maximum values of ${Ca}_{cyt}$ increase with $p_{GABA,prod}$, and the oscillation frequency rises as well. The model therefore predicts that GABA production affects not only the amplitude of cytosolic Ca^2+^ oscillations, but also their temporal organization. Higher GABA production is associated with larger-amplitude and higher-frequency calcium oscillations, whereas reduced GABA production leads to smaller amplitudes and lower oscillation frequencies.

Because GABA influences the system through more than one pathway, including intracellular metabolic effects on ATP production and interstitial signaling effects on Ca^2+^ influx, we next analyze these mechanisms separately. This allows us to distinguish the relative contributions of the metabolic and signaling actions of GABA to the modulation of Ca^2+^ oscillatory dynamics.

#### 3.1.1. Impact of GABA-Dependent ATP Production on Ca^2+^ Oscillations

**Figure 4:**
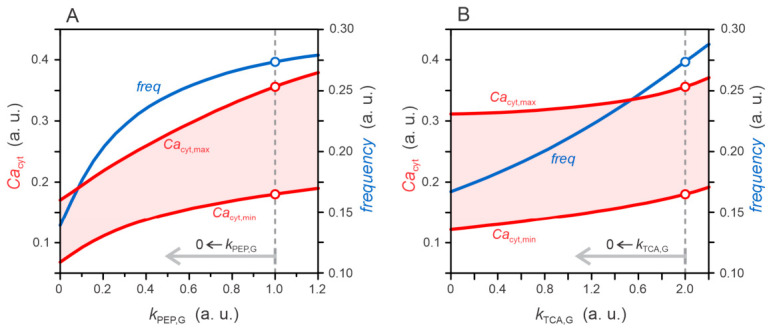
Effect of GABA-dependent ATP production on Ca^2+^ oscillations. (**A**) Dependence of the minimum and maximum values of cytosolic Ca^2+^ oscillations, ${Ca}_{cyt,min}$ and ${Ca}_{cyt,max}$, and oscillation frequency, freq, on the parameter $k_{PEP,G}$, which quantifies the GABA-dependent contribution to ATP production through the PEP-related branch. (**B**) Dependence of ${Ca}_{cyt,min}$, ${Ca}_{cyt,max}$, and freq on the parameter $k_{TCA,G}$, which determines the GABA-dependent contribution to ATP production through the TCA-related branch. Vertical dashed lines indicate the reference values of $k_{PEP,G}$ and $k_{TCA,G}$ used in the baseline model. Open circles mark the corresponding reference values of $Ca_{cyt,min}$, $Ca_{cyt,max}$, and freq at these parameter values.

In the analysis above, variation of the overall GABA production rate altered both intracellular GABA metabolism and interstitial GABA signaling. We next isolate the metabolic contribution of GABA and examine how GABA-dependent ATP production affects Ca^2+^ oscillations (Figure 4). For this purpose, GABA production itself is kept unchanged, whereas its contribution to ATP generation is selectively reduced through the parameters $k_{PEP,G}$ and $k_{TCA,G}$. As defined in Equations (13) and (14), these parameters determine how strongly GABA production enhances ATP generation through the PEP-related and TCA-related components, respectively. This approach allows us to assess how the intracellular metabolic action of GABA, independently of its receptor-mediated interstitial/paracrine effects, contributes to the modulation of Ca^2+^ oscillatory dynamics.

Figure 4A shows the effect of the parameter $k_{PEP,G}$, which determines the GABA-dependent contribution to ATP production through the PEP-related branch, on the properties of cytosolic Ca^2+^ oscillations. As $k_{PEP,G}$ decreases, corresponding to a progressive reduction of the GABA effect on this component of ATP production, both the minimum and maximum values of $Ca_{cyt}$ oscillations decrease, accompanied by a clear reduction in oscillation amplitude. The oscillation frequency also decreases, with the decline being most pronounced at lower values of $k_{PEP,G}$.

Figure 4B shows the corresponding effect of the parameter $k_{TCA,G}$, which determines the GABA-dependent contribution to ATP production through the TCA-related branch. In this case, decreasing $k_{TCA,G}$ likewise lowers both the minimum and maximum values of $Ca_{cyt}$ oscillations, whereas the oscillation amplitude remains comparatively stable over most of the parameter range. The oscillation frequency nevertheless decreases, with the effect becoming somewhat more pronounced at higher values of $k_{TCA,G}$.

Taken together, these results indicate that GABA-dependent ATP production in both metabolic branches contributes to the maintenance of higher cytosolic Ca^2+^ levels and higher oscillation frequencies. However, the PEP-related branch has a more prominent effect on oscillation amplitude, whereas the TCA-related branch primarily affects oscillation timing while leaving the amplitude relatively preserved.

To further explain the changes in Ca^2+^ oscillation amplitude and frequency shown in Figure 4, we next examine how selective blockade of the GABA-dependent contribution to ATP production reshapes the coupled dynamics of local ATP near $K_{ATP}$ channels and cytosolic Ca^2+^. These two variables are tightly interdependent, and their temporal interplay provides the mechanistic basis for the changes in Ca^2+^ oscillatory behavior observed in the model.

**Figure 5:**
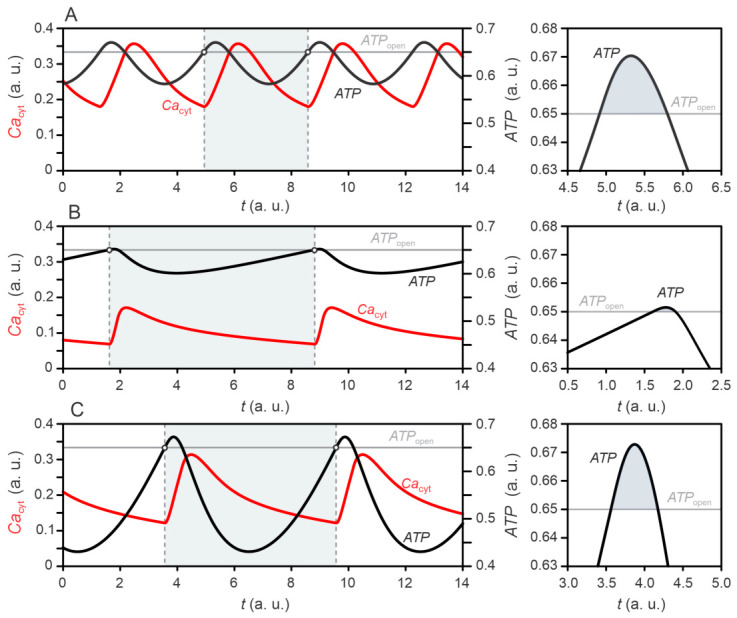
Effect of GABA-dependent ATP production on the coupled dynamics of local ATP and cytosolic Ca^2+^. (**A**) Reference model dynamics. (**B**) Dynamics after blocking the GABA-dependent contribution to ATP production through the PEP-related branch ($k_{PEP,G}=0$). (**C**) Dynamics after blocking the GABA-dependent contribution to ATP production within the TCA-related branch ($k_{TCA,G}=0$). In the left panels, black curves show the local ATP concentration near $K_{ATP}$ channels, and red curves show cytosolic calcium, ${Ca}_{cyt}$. The horizontal gray line indicates ${ATP}_{open}$, the threshold ATP level required to activate Ca^2+^ influx in the model. Vertical dashed lines mark the time points at which ATP crosses ${ATP}_{open}$; the interval between two successive crossings corresponds to one oscillatory cycle. The right panels show enlarged views of the ATP transients in the vicinity of the threshold-crossing regions.

Figure 5 shows the dynamics of local ATP near $K_{ATP}$ channels and cytosolic calcium, ${Ca}_{cyt}$, under reference conditions (Figure 5A), after blocking the GABA-dependent contribution to ATP production through the PEP-related branch **(**Figure 5B), and after blocking the corresponding contribution through the TCA-related branch (Figure 5C). Here, ${ATP}_{open}$ denotes the threshold ATP level required to activate the Ca^2+^ influx pathway in the model (Equation (10)). When ATP rises above this threshold, Ca^2+^ influx is initiated and ${Ca}_{cyt}$ begins to increase. The subsequent rise in cytosolic Ca^2+^ activates Ca^2+^-removal processes, which in turn reduce ${Ca}_{cyt}$ and terminate the calcium pulse. Thus, the timing and magnitude of ATP excursions relative to ${ATP}_{open}$ determine both the onset and the strength of the corresponding Ca^2+^ response.

When the GABA-dependent contribution to ATP production through the PEP-related branch is blocked ($k_{PEP,G}=0$), ATP generation during the cataplerotic phase becomes slower. This branch corresponds to the GABA-dependent support of the PEP cycle, which is most effective when ${Ca}_{cyt}$ is low. As a result, the local ATP concentration near $K_{ATP}$ channels rises more gradually, as reflected by the shallower ATP upstroke in Figure 5B. Consequently, ATP exceeds the threshold value ${ATP}_{open}$ only weakly, leading to a smaller Ca^2+^ influx and, therefore, to a lower peak of ${Ca}_{cyt}$. Because the Ca^2+^ rise remains reduced, the subsequent oxidative support of ATP production also remains limited. Together, these effects lead to both a lower amplitude and a lower frequency of Ca^2+^ oscillations.

When the GABA-dependent contribution to ATP production through the TCA-related branch is blocked ($k_{TCA,G}=0$), ATP production during the oxidative phase is reduced because the contribution of the GABA shunt to oxidative ATP production, mediated by the return of carbon from the GABA pool into the TCA cycle, is abolished. Under these conditions, the local ATP concentration declines more strongly during the active phase of the oscillation. During the following cataplerotic phase, ATP production increases again; however, because ATP starts from a lower level, more time is required to reach the threshold value ${ATP}_{open}$. This primarily prolongs the oscillation period and thus lowers the oscillation frequency. In contrast, the amplitude of the Ca^2+^ oscillations remains relatively preserved, because the late ATP rise still produces a sufficient overshoot above ${ATP}_{open}$ to trigger a pronounced Ca^2+^ influx, as shown in Figure 5C.

Taken together, the results in Figure 5 show that the GABA-dependent ATP-producing contribution through the PEP cycle is particularly important for generating a rapid and sufficiently large ATP rise during the cataplerotic phase, which in turn determines both the amplitude and the frequency of the subsequent Ca^2+^ oscillations. By contrast, the GABA-dependent contribution associated with the GABA shunt and oxidative TCA-cycle metabolism primarily supports ATP during the oxidative phase and therefore affects oscillation timing more strongly than oscillation amplitude.

#### 3.1.2. Impact of GABA-Dependent Ca^2+^ Influx on Ca^2+^ Oscillations

We next examine how interstitial GABA modulates Ca^2+^ entry into the cell and, consequently, the dynamics of cytosolic Ca^2+^ oscillations. In the model, this regulation acts through the influx term $J_{in}$, which describes Ca^2+^ entry from the extracellular space into the cytosol. As summarized in Equations (7)–(10), ${GABA}_{is}$ affects $J_{in}$ through two inhibitory mechanisms. First, it directly reduces the effective maximal Ca^2+^ influx rate by decreasing $g_{in}$. Second, it gradually lowers the effective channel activation variable x, thereby introducing a slower inhibitory feedback on Ca^2+^ entry. Together, these two actions represent the combined fast and delayed effects of interstitial GABA on Ca^2+^ influx and provide the basis for analyzing how interstitial GABA signaling reshapes Ca^2+^ oscillatory dynamics.

To quantify this effect, we introduce the dimensionless parameter $p_{GABA,is}$, which rescales the interstitial GABA signal according to

(20) $${GABA}_{is}\;:=p_{GABA,is}\cdot {GABA}_{is}.$$

The parameter $p_{GABA,is}$ ranges from 0 to 1 and therefore controls the strength of the ${GABA}_{is}$-dependent inhibition in Equations (8) and (10). A value of $p_{GABA,is}=0$ corresponds to a complete blockade of the interstitial GABA effect on Ca^2+^ influx, whereas $p_{GABA,is}=1$ preserves the full inhibitory contribution of ${GABA}_{is}$ used in the reference model.

**Figure 6:**
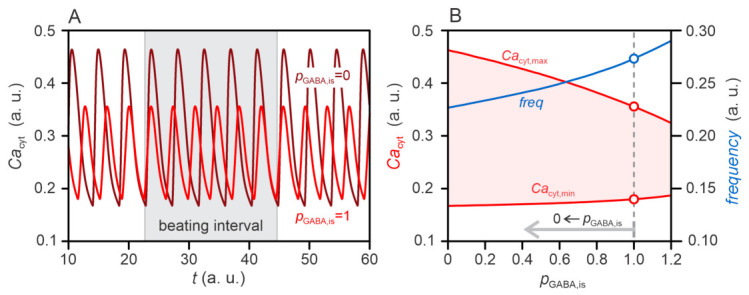
Effect of interstitial GABA on cytosolic Ca^2+^ oscillations. (**A**) Time courses of cytosolic calcium, ${Ca}_{cyt}$, shown for the reference interstitial GABA effect ($p_{GABA,is}=1$) and for complete blockade of this effect ($p_{GABA,is}=0$). The shaded region highlights the gradual phase divergence between the two oscillatory signals, giving rise to a slow beating interval. (**B**) Dependence of the minimum and maximum values of the calcium oscillations, ${Ca}_{cyt,min}$ and ${Ca}_{cyt,max}$, and the oscillation frequency, freq, on $p_{GABA,is}$. The vertical dashed line indicates the reference value $p_{GABA,is}=1$ used in the baseline model. Open circles denote the corresponding reference values of ${Ca}_{cyt,min}$, ${Ca}_{cyt,max}$, and freq.

The resulting changes in Ca^2+^ dynamics are shown in Figure 6. Figure 6A compares representative time courses of $Ca_{cyt}$ for the reference case ($p_{GABA,is}=1$) and for complete blockade of the interstitial GABA effect ($p_{GABA,is}=0$). Blocking the effect of interstitial GABA on $J_{in}$ leads to a slight increase in the amplitude of $Ca_{cyt}$ oscillations, consistent with the inhibitory action of ${GABA}_{is}$ on Ca^2+^ entry. In contrast, the oscillation frequency changes only modestly. This behavior is also evident in Figure 6A, where the two calcium traces remain very similar in shape but gradually develop a phase shift over time. The shaded region highlights the resulting slow beating interval, which arises from the small difference in oscillation frequency between the two conditions.

This behavior is quantified in Figure 6B, which shows the dependence of ${Ca}_{cyt,min}$, ${Ca}_{cyt,max}$, and oscillation frequency on $p_{GABA,is}$. As the strength of interstitial GABA signaling is reduced, both the minimum and maximum values of ${Ca}_{cyt}$ increase slightly, indicating a modest increase in oscillation amplitude. By contrast, the oscillation frequency changes only weakly over the entire parameter range. Thus, Figure 6B confirms that the primary effect of interstitial GABA on the single-cell oscillator is not a strong shift in intrinsic oscillation frequency, but rather a comparatively small modulation of Ca^2+^ influx that manifests mainly through subtle changes in oscillation amplitude and timing.

Although the isolated effect of interstitial GABA on Ca^2+^ influx produces only a modest change in oscillation frequency, it plays an important mechanistic role by adjusting oscillatory phase. This division of roles follows from the structure of the model and from the separate parameter analyses shown in Figure 4, Figure 5 and Figure 6. ATP dynamics enters the oscillator through the production and consumption terms in Equation (11), and the timing of ATP excursions relative to the activation threshold determines when Ca^2+^ influx is initiated. Consequently, changes in the GABA-dependent ATP-producing branches alter the time required for ATP to reach the threshold and therefore change the inter-peak interval and oscillation frequency. By contrast, interstitial GABA acts downstream of this metabolic rhythm by modulating the effective Ca^2+^ influx term and the associated activation variable (Equations (7)–(10)). When this signaling component is isolated, the ATP-driven rhythm is largely preserved, whereas the timing of Ca^2+^ entry is shifted, producing gradual phase divergence and beating rather than a major change in intrinsic frequency. Thus, within the present reduced model, ATP dynamics primarily sets the metabolic timescale of the oscillator, whereas interstitial GABA mainly provides phase adjustment of Ca^2+^ entry. This distinction is especially relevant in the coupled-cell setting, where moderate phase shifts in Ca^2+^ influx can facilitate entrainment between neighboring cells and thereby promote synchronization.

### 3.2. Intercellular GABA Couples and Entrains Ca^2+^ Oscillations in Two Beta Cells

We next examined whether the GABA-dependent modulation of Ca^2+^ influx, which in the single-cell analysis acted primarily as a phase-adjusting mechanism, is sufficient to promote entrainment and synchronization between two coupled beta cells. For this purpose, we used the two-cell model introduced in Section 2.2, in which each cell retains its own intracellular metabolic and Ca^2+^ dynamics, whereas coupling is mediated through the shared interstitial GABA signal ${GABA}_{is,avg}$. In this way, intracellular GABA oscillations generated by the metabolic subsystem are transformed into a common interstitial coupling signal that acts back on both cells and modulates Ca^2+^ influx in each of them.

**Figure 7:**
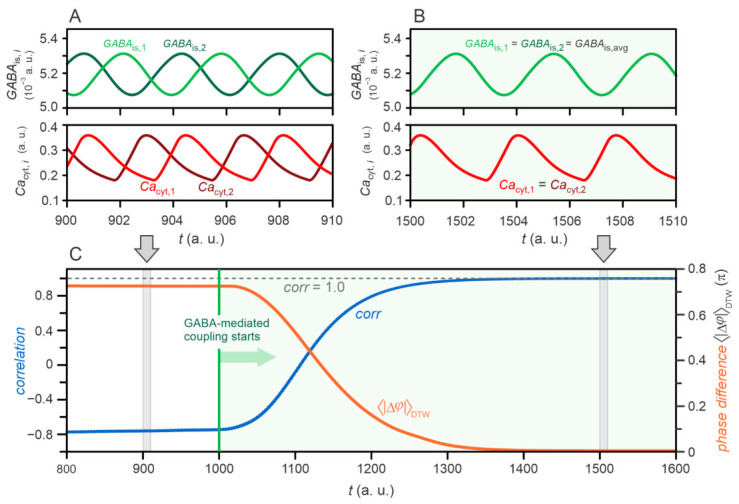
GABA-mediated intercellular coupling entrains Ca^2+^ oscillations in two beta cells. (**A**) Early transient regime before full entrainment. The upper panel shows the interstitial GABA signals of the two cells, ${GABA}_{is,1}$ and ${GABA}_{is,2}$, which are phase-shifted relative to each other. The lower panel shows the corresponding cytosolic Ca^2+^ signals, ${Ca}_{cyt,1}$ and ${Ca}_{cyt,2}$, which are also clearly phase-shifted, indicating that the two cells are not yet synchronized. (**B**) Late regime after GABA-mediated entrainment. The two interstitial GABA signals overlap and form a common signal, ${GABA}_{is,1}={GABA}_{is,2}={GABA}_{is,avg}$. The corresponding Ca^2+^ signals also overlap, ${Ca}_{cyt,1}={Ca}_{cyt,2}$, indicating effective synchronization of the two cells. (**C**) Time evolution of the exponentially weighted moving Pearson correlation coefficient, corr, calculated between the two Ca^2+^ signals following Pozzi et al. [28]. The orange curve shows the mean absolute phase difference inspired by dynamic time warping (DTW), ${\langle \left|∆\varphi \right|\rangle }_{DTW}$, plotted on the right *y*-axis and expressed in units of π. This phase-difference measure was estimated from the time shift that minimized the distance between the normalized Ca^2+^ signals. A value of ${\langle \left|∆\varphi \right|\rangle }_{DTW}=0$ indicates phase alignment, whereas larger values indicate increasing phase mismatch. After GABA-mediated coupling is switched on, *corr* increases toward 1, while ${\langle \left|∆\varphi \right|\rangle }_{DTW}$ decreases toward 0, confirming progressive synchronization and phase entrainment of the two Ca^2+^ oscillators. The gray vertical bands mark the time intervals shown in panels A and B, while the arrows indicate their correspondence with the correlation trace. The green vertical line denotes the onset of GABA-mediated intercellular coupling, and the light green shaded region indicates the time interval after coupling is switched on.

The corresponding dynamics are shown in Figure 7. At the beginning of the simulation (Figure 7A), the two cells exhibit a clear phase difference in their cytosolic Ca^2+^ oscillations, indicating that they are initially not entrained. Consistent with this, the two interstitial GABA signals are also phase-shifted during the initial regime. After the onset of GABA-mediated coupling, both cells begin to sense the shared interstitial GABA signal $GABA_{is,avg}$, which progressively reduces the phase difference between the two oscillators by jointly modulating Ca^2+^ influx. To make the late stage of this process more visible, Figure 7 contains a break in the time axis. In the later regime shown in Figure 7B, the two interstitial GABA signals overlap and form a common oscillatory signal, while the corresponding cytosolic Ca^2+^ traces also overlap almost completely, indicating effective entrainment and near-complete synchronization.

As an additional quantitative confirmation of this transition, synchronization was characterized by a time-dependent Pearson correlation coefficient, corr, calculated over an exponentially weighted moving window, following the general approach of Pozzi et al. [28]. Since the Pearson correlation coefficient provides a compact measure of the overall similarity between the two Ca^2+^ traces but does not explicitly quantify their temporal phase alignment, we additionally calculated a mean absolute phase difference inspired by dynamic time warping (DTW), denoted as ${\langle \left|∆\varphi \right|\rangle }_{DTW}$. This measure was obtained by estimating the time shift that minimized the distance between the normalized Ca^2+^ signals, following the general rationale of dynamic time warping for time-series alignment [29]. Thus, whereas the Pearson coefficient quantifies the overall similarity of the two oscillatory signals, ${\langle \left|∆\varphi \right|\rangle }_{DTW}$ directly reflects their phase mismatch, with values approaching 0° indicating phase alignment. In this type of synchronization analysis, it is also meaningful to examine oscillation-period consistency. In the present simulation, however, the two Ca^2+^ oscillators were initially phase shifted but had the same intrinsic oscillation period, and this period was preserved after GABA-mediated coupling was switched on. Therefore, the synchronization transition is quantified here by the complementary behavior of the Pearson correlation coefficient and the DTW-inspired phase-difference measure. As shown in Figure 7C, the correlation is initially low or negative, consistent with the phase-shifted oscillations in Figure 7A, whereas ${\langle \left|∆\varphi \right|\rangle }_{DTW}$ is initially high. After GABA-mediated coupling is switched on, the correlation rises progressively toward values close to 1, while ${\langle \left|∆\varphi \right|\rangle }_{DTW}$ decreases toward 0°, indicating convergence of the two Ca^2+^ oscillators to a common rhythm and phase.

These results show that even a relatively modest GABA-dependent modulation of Ca^2+^ influx is sufficient to coordinate oscillatory beta-cell activity when transmitted through the interstitial space. In mechanistic terms, ATP-dependent metabolic dynamics primarily determine the intrinsic oscillatory rhythm of each cell, whereas intercellular GABA provides the coupling signal that adjusts phase and gradually aligns the two oscillators. Thus, the model supports a division of roles in which metabolism sets the intrinsic pace of the oscillations, while interstitial GABA-mediated signaling enables entrainment and synchronization between neighboring cells. This interpretation is consistent with the experimental view that beta-cell-derived GABA can function as an intercellular coordinating signal within the islet [14].

To further evaluate the physiological relevance of this coupling mechanism, we next examined how strongly the parameters of the two cells may differ while synchronization is still maintained. This question is important because beta cells within the islet are intrinsically heterogeneous and therefore do not share identical metabolic, signaling, or excitability properties. In the model, such physiological variability is represented by differences in selected parameter values between the two cells. We therefore asked whether GABA-mediated coupling remains sufficiently robust to entrain cells that differ in their intrinsic oscillatory characteristics. Rather than analyzing all model parameters, we selected a representative set of nine parameters according to their mechanistic roles in the system. Specifically, the selected set includes parameters describing the metabolic component of the model, namely GABA production, the PEP-related and TCA-related contributions to ATP generation, and the two ATP-consumption terms; parameters representing interstitial GABA action on Ca^2+^ influx, including the overall strength of the interstitial GABA signal and the fast and slow inhibitory coupling pathways; and a parameter characterizing intrinsic cellular excitability through the ATP threshold for activation of Ca^2+^ influx. In this way, the selected set spans the principal determinants of the intrinsic oscillatory rhythm, the strength of intercellular GABA-mediated phase adjustment, and the baseline responsiveness of the Ca^2+^-influx pathway. Table 1 summarizes the corresponding synchronization ranges and thereby illustrates not only the robustness of the model itself, but also the capacity of GABA-mediated entrainment to synchronize metabolically and functionally non-identical beta cells.

In non-identical cells, GABA-mediated coupling does not necessarily induce complete in-phase synchronization of the two Ca^2+^ signals (${\langle \left|∆\varphi \right|\rangle }_{DTW}\to 0$ and corr→1), because the cells represent non-identical oscillators with slightly different intrinsic frequencies and intrinsic dynamics. Thus, coupling does not fully equalize their signals, but rather stabilizes their relative phase, allowing the cells to oscillate with a common rhythm while maintaining a finite phase difference (${\langle \left|∆\varphi \right|\rangle }_{DTW}>0$ and corr<1). Table 1 shows the ranges of selected parameters in the second cell relative to the first cell for which GABA-mediated coupling still supports stable phase locking. In this analysis, stable phase locking was identified when the DTW-inspired mean absolute phase difference remained below ${\langle \left|∆\varphi \right|\rangle }_{DTW}\leq 0.2\pi$, corresponding to a Ca^2+^ signal phase lag of no more than 10% of the oscillation period. This criterion was also consistent with a moving Pearson correlation coefficient of corr>0.7.

Column A of Table 1 shows the synchronization ranges obtained for the reference strength of the interstitial GABA signal ($p_{GABA,is}=1.0$). To assess how stronger interstitial GABA action influences synchronization between the two cells, Column B shows the corresponding ranges for the increased value ($p_{GABA,is}=1.2$). Comparison of Columns A and B shows that increasing the influence of interstitial GABA generally broadens the range of parameter mismatch over which stable phase locking is still achieved (${\langle \left|∆\varphi \right|\rangle }_{DTW}\leq 0.2\pi$, corr>0.7). This indicates that a stronger shared GABA signal more effectively compensates for differences in the intrinsic properties of the two cells and thereby enhances the robustness of synchronization.

A notable exception is the parameter $k_{PEP,G}$, for which increasing $p_{GABA,is}$ reduces the upper limit of the synchronization range. A positive deviation of $k_{PEP,G}$ in the second cell enhances the PEP-related contribution to ATP production, causing local ATP to rise more rapidly and to reach the threshold for Ca^2+^ influx earlier. As a result, the corresponding Ca^2+^ pulse is advanced in time relative to that of the first cell. This interpretation is consistent with Figure 5A,B, which shows that the PEP-related branch strongly affects the rate of ATP rise and the timing of threshold crossing. Moreover, Figure 4A indicates that increasing $k_{PEP,G}$ above its reference value ($k_{PEP,G}=1$) changes oscillation frequency less strongly than decreasing it below the reference value, which explains the relatively broad positive synchronization range under reference coupling. When interstitial GABA signaling is strengthened ($p_{GABA,is}=1.2$), however, the delayed GABA-dependent inhibition of Ca^2+^ influx is also enhanced. Under these conditions, larger positive deviations of $k_{PEP,G}$ lead to a less favorable balance between earlier ATP-dependent triggering and stronger delayed inhibitory feedback, thereby narrowing the upper bound of the synchronization range. In other words, stronger interstitial GABA generally improves phase locking, but in the case of excessive enhancement of the PEP-related ATP-producing branch it can also accentuate the timing mismatch that limits stable entrainment.

The magnitude of the interstitial GABA signal also deserves specific consideration in the interpretation of the synchronization ranges. In the model, interstitial GABA was assumed to be approximately two orders of magnitude lower than the intracellular GABA level, as represented by the scaling factor $r_{G}=0.01$. However, physiological interstitial GABA concentrations in pancreatic islets remain uncertain. The results in Column A of Table 1 show that the parameter $r_{G}$ can be substantially varied in one of the two cells while stable phase locking is still maintained. The apparently narrower positive ranges for $r_{G}$ in Columns B and C should be interpreted with caution, because in these cases the overall influence of interstitial GABA is already increased ($p_{GABA,is}=1.2$). Thus, the same change in $r_{G}$ produces stronger effective inhibitory feedback on Ca^2+^ influx. Conversely, the admissible range of $r_{G}$ would be expected to broaden if the coupling constants $k_{in,G}$ (Equation (8)) and $k_{x,G}$ (Equation (10)), which determine the strength of GABA-dependent inhibition of Ca^2+^ influx, were reduced. These results indicate that the model predictions do not depend on a narrowly fixed interstitial GABA concentration. Rather, synchronization is governed by the effective strength of GABA-mediated feedback, suggesting that stable phase locking can be maintained over a broad range of physiologically plausible interstitial GABA levels.

The parameter differences summarized in Table 1 are also associated with changes in the intrinsic oscillation period of the individual uncoupled cells. This follows directly from the single-cell analyses, where changes in GABA production, $p_{GABA,prod}$, modified the Ca^2+^ oscillation frequency (Figure 3C), and changes in the GABA-dependent ATP-production parameters $k_{PEP,G}$ and $k_{TCA,G}$ likewise affected oscillation frequency (Figure 4). Similarly, changes in the strength of interstitial GABA action slightly altered the oscillation frequency through feedback on Ca^2+^ influx (Figure 6B). Thus, parameter mismatch between the two cells can generate not only phase differences, but also small differences in their intrinsic oscillation periods. In the synchronized regimes reported in Table 1, GABA-mediated coupling overcomes these differences by stabilizing phase locking between the two Ca^2+^ signals. Once phase locking is established, the coupled cells oscillate with a common effective period, although this period may differ slightly from the intrinsic period of either uncoupled cell because the shared interstitial GABA signal, ${GABA}_{is,avg}$, feeds back on Ca^2+^ influx in both cells. Therefore, the synchronization ranges in Table 1 reflect not only high correlation and small phase mismatch, but also oscillation-period consistency.

To further extend the analysis, we asked whether synchronization could be reinforced not only by paracrine GABA-mediated coupling, but also by a weak effective electrical interaction between the cells. This question is physiologically relevant because beta cells within the islet are connected not only through paracrine signaling, but also through direct electrical coupling via gap junctions, predominantly formed by connexin-36 (Cx36) channels. Experimental studies have shown that Cx36-mediated gap-junction coupling is important for the synchronization of glucose-induced Ca^2+^ oscillations and pulsatile insulin secretion in beta cells and intact islets [30,31,32]. In addition, experimental and computational studies have demonstrated that gap-junction coupling, together with beta-cell heterogeneity, can shape coordinated Ca^2+^ wave propagation and population-level synchronization within the islet [33]. Because the present model does not include an explicit equation for membrane potential, we represented this effect phenomenologically by adding an additional term to Equation (8), which defines the conductance of Ca^2+^ influx,

(21) $$g_{in,i}=g_{in,0}-k_{G}{GABA}_{is,avg}+k_{el}({Ca}_{cyt,j}-{Ca}_{cyt,i}),$$

where $k_{el}$ determines the strength of weak effective electrical coupling. This term does not represent direct diffusion of Ca^2+^ between cells; rather, it provides an effective description of how electrical coupling can influence cellular excitability and, consequently, voltage-dependent Ca^2+^ entry. The results of this case are summarized in Column C of Table 1 ($p_{GABA,is}=1.2$, $k_{el}=1$). Comparison with Column B shows that the addition of weak electrical coupling further broadens the ranges of all selected parameters for which stable phase locking is maintained (corr>0.7). This indicates that weak electrical coupling can complement GABA-mediated synchronization and further increase the robustness of coordinated activity between non-identical cells. This result should not be interpreted as a quantitative simulation of Cx36-mediated gap-junction currents. Rather, it shows that, within the present reduced framework, an additional weak electrical influence on excitability can act cooperatively with interstitial GABA-mediated phase adjustment. Thus, GABA-mediated coupling and electrical coupling should not be viewed as mutually exclusive synchronization mechanisms. Instead, the model suggests that interstitial GABA can provide a paracrine phase-adjusting signal, whereas electrical coupling can further stabilize and broaden the range over which phase locking is maintained.

Viewed together, the results summarized in Table 1 show that GABA-mediated intercellular coupling is sufficiently robust to entrain beta cells even in the presence of physiologically plausible heterogeneity, and that this robustness can be further enhanced by weak effective electrical coupling. The model therefore supports the view that interstitial GABA can act as an effective coordinating signal that aligns the phases of non-identical beta-cell oscillators and promotes synchronized Ca^2+^ activity at the islet level, while electrical coupling provides an additional stabilizing influence on this process. This interpretation is consistent with experimental evidence showing that pulsatile GABA release from beta cells can contribute to the coordination of islet activity [14], with the broader view that interstitial GABA participates in receptor-mediated auto/paracrine regulation of islet function [15], and with recent findings that endogenous beta-cell-derived GABA is required for proper Ca^2+^ oscillation dynamics and insulin secretion [17].

## 4. Discussion

The present study provides a mechanistic framework linking intracellular GABA metabolism to both single-cell Ca^2+^ oscillations and intercellular synchronization in pancreatic beta cells. By combining the previously developed DAM framework [18] with explicit equations for cytosolic Ca^2+^, ER Ca^2+^, ATP, and GABA-dependent regulation of Ca^2+^ influx, we were able to connect metabolic, signaling, and collective aspects of beta-cell function within a single dynamical model. In this way, the model extends earlier conceptual work on the metabolic role of the GABA shunt in beta cells [8,12,13] toward an explicit explanation of how GABA shapes Ca^2+^ oscillatory dynamics and coordinated beta-cell behavior.

A central result of the study is that reduced GABA production leads to lower-amplitude and lower-frequency Ca^2+^ oscillations. This prediction is in good qualitative agreement with the recent experiments of Ferreira et al. [17], who showed that endogenous beta-cell-derived GABA is required for properly shaped Ca^2+^ oscillations and normal insulin secretion. In the present model, this phenotype is not imposed phenomenologically, but emerges from the coupled effects of reduced intracellular GABA metabolism and weaker interstitial GABA signaling. Thus, the model provides a mechanistic explanation for how impaired endogenous GABA availability can alter both the magnitude and timing of beta-cell Ca^2+^ signals.

The analysis further shows that the metabolic action of GABA is best understood within the dual-anaplerotic logic of the DAM. In this framework, GABA contributes to ATP production in two related but functionally distinct ways. First, through the GABA shunt, it supports oxidative ATP production during the oxidative phase by returning carbon from the GABA pool into the TCA cycle. Second, by increasing the effective carbon volume available for subsequent cataplerosis, it indirectly promotes the PEP cycle and thereby strengthens local ATP production during the cataplerotic phase. The model indicates that this PEP-related contribution is especially important for generating a rapid and sufficiently large ATP rise near K_ATP_ channels, which in turn is crucial for both the amplitude and the frequency of the ensuing Ca^2+^ oscillations. By contrast, the oxidative TCA-related contribution primarily stabilizes ATP during the active phase and therefore affects oscillation timing more strongly than oscillation amplitude. This interpretation is consistent with the broader view that GABA metabolism is functionally integrated with beta-cell bioenergetics and insulin secretion [8,13,23,24].

A second important result is that interstitial GABA signaling, when considered in isolation, exerts only a modest effect on the intrinsic oscillation frequency of a single cell, but plays a key role in phase adjustment. In the model, this interstitial/paracrine action is represented by a fast inhibitory effect on the effective Ca^2+^-influx term and a slower inhibitory effect mediated through the regulatory variable x. Physiologically, these two components correspond to a simplified description of ionotropic and metabotropic GABAergic actions, respectively, and are consistent with the current view that beta-cell-derived GABA acts through both auto/paracrine receptor-mediated pathways and metabolic coupling [15,16,17]. The present results therefore suggest a functional division of labor between the two major GABA actions in the model: ATP dynamics primarily determine the intrinsic oscillatory rhythm, whereas interstitial GABA-dependent modulation of Ca^2+^ influx primarily adjusts oscillatory phase.

This distinction becomes particularly important at the multicellular level. Once two cells are coupled through the shared interstitial GABA signal, even a relatively small GABA-dependent phase adjustment of Ca^2+^ influx is sufficient to entrain the two oscillators and drive them toward synchronized activity. Importantly, this synchronization persists over finite ranges of parameter mismatch between the two cells, which is physiologically relevant because beta cells within the islet are intrinsically heterogeneous in their metabolic activity, signaling properties, and excitability. The model therefore supports the view that interstitial GABA can act as an effective coordinating signal that aligns the phases of non-identical beta-cell oscillators. In addition, our analysis shows that weak effective electrical coupling can further broaden the parameter ranges over which stable phase locking is maintained, indicating that paracrine GABA signaling and electrical interactions may act in a complementary manner to stabilize coordinated beta-cell activity. This interpretation is consistent with experimental evidence that pulsatile GABA release from beta cells can contribute to the coordination of islet activity [14], with the broader view that interstitial GABA participates in receptor-mediated auto/paracrine regulation of islet function [15], with the observation that endogenous GABA is required for proper Ca^2+^ oscillation dynamics [17], and with studies showing that Cx36-mediated coupling supports synchronized beta-cell Ca^2+^ activity and pulsatile insulin secretion [30,31,32,33].

These findings may also have implications for beta-cell dysfunction, diabetes, and islet remodeling. Islet GABA levels and GABA-related signaling have been reported to be altered in diabetes, and impaired GABA metabolism has been proposed to contribute to beta-cell dysfunction and degradation in type 2 diabetes [13,15]. In addition, beta-cell-specific loss of GAD65 and GAD67 disrupts endogenous islet GABA, alters Ca^2+^ oscillatory activity, and impairs insulin secretion [17]. Within the framework of the present model, such alterations would be expected to affect beta-cell function at two coupled levels: metabolically, by weakening GABA-shunt-dependent support of ATP dynamics, and paracrinely, by reducing interstitial GABA-dependent phase adjustment between cells. Both effects could contribute to impaired pulsatile insulin secretion and loss of coordinated islet activity during beta-cell dysfunction. Experimental studies showing that exogenous GABA can support beta-cell survival, proliferation, and glucose homeostasis in diabetic or human-islet transplantation models further suggest that GABA-dependent pathways may be relevant not only for acute oscillatory regulation, but also for beta-cell adaptation and remodeling [34,35]. However, the present model addresses short-term metabolic and signaling dynamics, not beta-cell proliferation, immune modulation, or structural remodeling. Future extensions should therefore examine how altered GABA production, GABA-shunt activity, and interstitial GABA signaling interact with diabetic stress, changing endocrine cell composition, and remodeling of islet network architecture.

From a modeling perspective, the present framework does not aim to replace detailed electrophysiological models of beta-cell activity, but rather to complement them. Previous mathematical studies have provided important insight into bursting dynamics, the interaction between metabolic and electrical oscillations, oscillations in K_ATP_ conductance, phantom bursting, and the behavior of coupled and multicellular islet models [22,36,37,38,39,40]. Our model adds to this line of work by explicitly incorporating intracellular GABA dynamics, GABA-dependent ATP production, delayed interstitial GABA signaling, and a phenomenological weak electrical-coupling term into a minimal Ca^2+^–ATP oscillatory framework. In this sense, it can be viewed as a metabolically and paracrinely enriched module that captures how paracrine GABA signaling and electrical interactions may jointly contribute to beta-cell coordination, and that could in future work be integrated with more detailed membrane-potential-based models of beta-cell electrophysiology and islet network behavior.

Importantly, the weak electrical-coupling term included here should be interpreted only as an effective representation of the stabilizing influence of electrical communication on Ca^2+^ entry and excitability, not as a mechanistic model of Cx36-mediated gap-junction currents. Experimental and computational studies have clearly established that gap-junctional coupling is a major determinant of coordinated beta-cell activity and islet-level synchronization [30,31,32,33]. The present model does not challenge this view. Instead, it addresses a complementary question: whether DAM-derived GABA dynamics and delayed interstitial GABA signaling can provide an additional phase-adjusting mechanism that supports synchronization between non-identical beta-cell oscillators. Our simulations suggest that GABA-mediated phase alignment can occur in the reduced two-cell system and that weak effective electrical coupling further increases its robustness. However, the relative quantitative contribution of GABA-mediated paracrine coupling versus Cx36-mediated electrical coupling under physiological conditions cannot be determined from the present model and will require future models with explicit membrane potential dynamics and gap-junction currents.

The model also has several limitations that are important for its biological interpretation. Membrane potential and individual ionic currents are not described explicitly, and the effects of ${GABA}_{A}$ and ${GABA}_{B}$ receptors are represented phenomenologically through effective modulation of Ca^2+^ influx and the slow regulatory variable, consistent with the view that interstitial GABA can modulate beta-cell Ca^2+^ dynamics through receptor-mediated signaling [15,17]. This reduction is appropriate for the present purpose, which is to analyze slow GABA-dependent changes in Ca^2+^-oscillation amplitude, frequency, phase adjustment, and synchronization, rather than receptor-specific kinetics or fast electrical activity. However, it also means that the model cannot distinguish the separate quantitative contributions of ${GABA}_{A}$- and ${GABA}_{B}$-mediated mechanisms, does not reproduce detailed membrane-potential waveforms or fast electrical bursting, and does not resolve receptor-specific chloride conductance, Gi/o signaling, cAMP dynamics, or downstream channel regulation. Therefore, the biological conclusions should be interpreted at the level of net interstitial GABA-dependent modulation of Ca^2+^ influx and intercellular phase coordination, not as predictions of receptor-specific electrical or biochemical kinetics.

Likewise, the interstitial GABA signal is represented by a simple delayed relation to cytosolic GABA, and the present multicellular analysis is restricted to a pair of coupled cells. The two-cell model should therefore be interpreted as a minimal coupling motif rather than as a direct representation of the full islet. Pancreatic islets contain large heterogeneous cell populations, including beta cells together with other endocrine cell types, and their collective activity is shaped by spatial organization, gap-junctional electrical coupling, paracrine interactions, vascular architecture, and cell-to-cell variability. The present two-cell formulation cannot capture these network-level features and should not be used to make quantitative predictions about whole-islet synchronization. Its purpose is more limited: to test whether DAM-derived intracellular GABA dynamics and delayed interstitial GABA signaling are, in principle, sufficient to phase-lock two non-identical beta-cell oscillators.

It should also be emphasized that the separation between ATP-dependent rhythm setting and GABA-dependent phase adjustment is a model-based mechanistic interpretation within the present reduced framework. In more complete beta-cell models, membrane-potential dynamics, gap-junction currents, receptor-specific GABA_A_/GABA_B_ signaling, cAMP pathways, and other modulators may also contribute to both frequency control and phase coordination. Thus, the present model identifies a possible local GABA-dependent coupling mechanism, but does not by itself establish the relative contribution of this mechanism within the full multicellular islet. The parameter ranges summarized in Table 1 support this interpretation by showing that GABA-mediated phase locking is maintained over finite ranges of several key parameters, rather than only at a single tuned parameter set. However, this analysis should be interpreted as a representative one-parameter-at-a-time robustness assessment, not as a formal global sensitivity analysis. A full variance-based or sampling-based global sensitivity analysis would require a broader parameter-space exploration and is left for future work, particularly because several parameters related to interstitial GABA signaling, receptor-dependent feedback, and effective electrical coupling are only weakly constrained experimentally.

Future extensions should therefore combine the present GABA-centered metabolic framework with detailed electrophysiological descriptions, explicit receptor kinetics, gap-junction currents, spatially organized multicellular islet architectures, and interactions between beta cells and other endocrine cell types. Such models could incorporate the present GABA-dependent mechanism as a modular component and quantify more explicitly how GABA metabolism, membrane excitability, electrical connectivity, paracrine signaling, and distributed islet synchronization interact under physiologically realistic conditions [19,38,40].

## 5. Conclusions

The present study identifies GABA as a dual regulator of pancreatic beta-cell oscillatory dynamics. Metabolically, GABA-shunt-related fluxes support local ATP dynamics through PEP-related and TCA-related ATP-producing branches, thereby shaping the amplitude and frequency of Ca^2+^ oscillations. Paracrinely, delayed interstitial GABA signaling modulates Ca^2+^ influx and provides a phase-adjusting mechanism that can promote entrainment and synchronization between non-identical beta-cell oscillators. The model further suggests that GABA-mediated phase adjustment can act together with weak effective electrical coupling to increase the robustness of synchronized activity in heterogeneous beta-cell populations.

The model reproduces key qualitative features observed under GABA-deficient conditions and suggests that impaired GABA production may affect beta-cell function at both the metabolic and intercellular coordination levels. Although the present formulation is intentionally reduced and does not replace detailed electrophysiological or multicellular islet models, it provides a mechanistic framework linking GABA metabolism, local ATP dynamics, Ca^2+^ signaling, and beta-cell synchronization. These findings support the view that GABA is not only a metabolic intermediate or local signaling molecule, but also a dynamic coordinator of beta-cell oscillatory activity, with potential relevance for beta-cell dysfunction, diabetes, and future models of islet remodeling.

## Figures and Tables

**Table 1:** Representative synchronization ranges for selected model parameters in two non-identical beta cells. The ranges indicate how much the parameter value in the second cell can deviate from the reference value of the first cell while stable phase locking is still maintained. Stable phase locking was defined by a dynamic time warping (DTW)-inspired mean absolute phase difference satisfying ${\langle \left|∆\varphi \right|\rangle }_{DTW}\leq 0.2\pi$, corresponding to a Ca^2+^ signal phase lag of no more than 10% of the oscillation period. This condition was also consistent with a moving Pearson correlation coefficient corr>0.7. Asterisks (*) indicate parameter boundaries at which the numerical integration terminated due to numerical instability, although the synchronization criterion remained satisfied up to that boundary. (A) Reference GABA-mediated coupling, $p_{GABA,is}=1.0$, without electrical coupling, $k_{el}=0$. (B) Increased interstitial GABA influence, $p_{GABA,is}=1.2$, without electrical coupling, $k_{el}=0$. (C) Increased interstitial GABA influence combined with weak effective electrical coupling, $p_{GABA,is}=1.2$, $k_{el}=1$.

			A		B		C	
Parameter	Equation	Ref. Val.	−∆p%	+∆p%	−∆p%	+∆p%	−∆p%	+∆p%
$p_{GABA,prod}$	(19)	1	−3%	3%	−9%	+77% *	−16%	+78% *
$p_{GABA,is}$	(20)	1 (1.2)	−3%	4%	−5%	+22%	−11%	+23%
$r_{G}$	(17)	0.01	−99% *	+50% *	−99% *	+28% *	−100%	+25% *
$k_{in,G}$	(8)	500	−9%	+9%	−15%	+22%	−36%	+88%
$k_{x,G}$	(10)	100	−5%	+5%	−7%	+27%	−18%	+28%
$k_{PEP,G}$	(13)	1	−10%	+104%	−44%	+23%	−48%	+47%
$k_{TCA,G}$	(14)	2	−2%	+2%	−6%	+7%	−11%	+32%
$k_{pump}$	(15)	1.5	−1%	+1%	−5%	+3%	−7%	+5%
$k_{use}$	(16)	0.1	−13%	+9%	−19%	+82%	−34%	+91%
${ATP}_{open}$	(10)	0.65	−2%	+2%	−3%	+3%	−6%	+6%

## Data Availability

The original contributions presented in this study are included in the article. Further inquiries can be directed to the corresponding author.

## Abbreviations

The following abbreviations are used in this manuscript:

a.u.	arbitrary units
αKG	α-ketoglutarate
ADP	adenosine diphosphate
ATP	adenosine triphosphate
ATP_open_	threshold ATP level required to activate Ca^2+^ influx
ATPase	ATP-consuming Ca^2+^ pumps
cAMP	cyclic adenosine monophosphate
*Ca*_cyt_	cytosolic calcium
*Ca*_ER_	endoplasmic reticulum calcium
CDI	Ca^2+^-dependent inactivation
CICR	Ca^2+^-induced Ca^2+^ release
Cl^−^	chloride ion
*corr*	Pearson correlation coefficient
Cx36	connexin-36
DAM DTW	Dual Anaplerotic Model dynamic time warping
ER	endoplasmic reticulum
ETC	electron transport chain
FADH_2_	reduced flavin adenine dinucleotide
Fum	fumarate
*freq*	oscillation frequency
GABA	γ-aminobutyric acid
${GABA}_{A}$	${GABA}_{A}$ receptor
${GABA}_{B}$	${GABA}_{B}$ receptor
GABA_cyt_	cytosolic GABA
GABA_is_	interstitial GABA
GABA_is,avg_	average interstitial GABA signal shared by coupled cells
GAD	glutamic acid decarboxylase
GAD65	65-kDa isoform of glutamic acid decarboxylase
GAD67	67-kDa isoform of glutamic acid decarboxylase
Gi/o	inhibitory G protein pathway
IOM	Integrated Oscillator Model
K_ATP_	ATP-sensitive potassium channel
NADH	reduced nicotinamide adenine dinucleotide
OAA	oxaloacetate
OxPhos	oxidative phosphorylation
PC	pyruvate carboxylase
PDH	pyruvate dehydrogenase
PEP	phosphoenolpyruvate
${\langle \left|∆\varphi \right|\rangle }_{DTW}$	phase difference inspired by dynamic time warping
Pyr	pyruvate
SERCA	sarco/endoplasmic reticulum Ca^2+^-ATPase
Suc	succinate
TCA	tricarboxylic acid cycle
TauT	taurine transporter
VGCC	voltage-gated calcium channel
VRAC	volume-regulated anion channel
